# A Blue Spectral Shift of the Hemoglobin Soret Band Correlates with the Age (Time Since Deposition) of Dried Bloodstains

**DOI:** 10.1371/journal.pone.0012830

**Published:** 2010-09-20

**Authors:** Erin K. Hanson, Jack Ballantyne

**Affiliations:** 1 National Center for Forensic Science, Orlando, Florida, United States of America; 2 Department of Chemistry, University of Central Florida, Orlando, Florida, United States of America; University Paris Diderot-Paris 7, France

## Abstract

The ability to determine the time since deposition of a bloodstain found at a crime scene could prove invaluable to law enforcement investigators, defining the time frame in which the individual depositing the evidence was present. Although various methods of accomplishing this have been proposed, none has gained widespread use due to poor time resolution and weak age correlation. We have developed a method for the estimation of the time since deposition (TSD) of dried bloodstains using UV-VIS spectrophotometric analysis of hemoglobin (Hb) that is based upon its characteristic oxidation chemistry. A detailed study of the Hb Soret band (λmax = 412 nm) in aged bloodstains revealed a blue shift (shift to shorter wavelength) as the age of the stain increases. The extent of this shift permits, for the first time, a distinction to be made between bloodstains that were deposited minutes, hours, days and weeks prior to recovery and analysis. The extent of the blue shift was found to be a function of ambient relative humidity and temperature. The method is extremely sensitive, requiring as little as a 1 µl dried bloodstain for analysis. We demonstrate that it might be possible to perform TSD measurements at the crime scene using a portable low-sample-volume spectrophotometer.

## Introduction

Current forensic biochemistry analytical technologies permit a significant amount of individual-specific genetic information to be obtained from a biological stain found at a crime scene [1]. New bio-analytical methods are being developed to determine the body fluid or tissue origin of a biological stain using RNA profiling (rather than conventional serological testing), as well as to predict the stain donor's physical characteristics such as eye, hair and skin color, bio-ancestry, biological age and facial features [2]–[15]. The novel evidence obtained from these approaches can aid law enforcement investigators in cases where there are no known suspects and thus constitutes a ‘genetic eyewitness’ description of the donor of a biological fluid that is not constrained or biased by human recollection or subjective accounts. However, additional probative information of a molecular genetic nature that does not relate to genetic individualization may also be present in dried stains. An example would be the ability to determine the time since deposition (TSD) of biological stains and is the subject of the present work.

The establishment of a time line of events in criminal offenses is often limited to eyewitness or victim accounts. If the crime involves murder it is sometimes possible, using various pathological cues provided by the corpse, to determine an approximate time of death. However, many criminal investigations do not include eyewitnesses or bodies for time of commission determinations although forensic evidence found at crime scenes is often in the form of dried biological stains or tissues. The problem is that few reliable and accurate methods exist to approximate the time of deposition of these dried biological stains [16]–[26]. Many of the methods developed to estimate an approximate age of a bloodstain have focused on deteriorative changes to the visible spectrum of hemoglobin (Hb) over time [17]–[19], [21]–[24]. For example, one such method used the α-chain to heme ratio determined by HPLC [19]. A linear decrease in the α-chain/heme peak area ratio was observed, on a logarithmic scale, as stain age increased. In a subsequent study, a peak designated as “X” was detected only in aged stains, and the area of this peak increased as the age of the stain increased [20]. Other studies have also used HPLC analysis of Hb to estimate TSD [17], [23], [24]. While these studies demonstrated some correlation between the age of a stain and structural changes to the Hb molecule, the reported methods provided inadequate resolution for the time intervals (i.e. hours, days, weeks and months) important in forensic analysis. Moreover the previous studies did not consider in detail the effect of important potential variables such as ambient temperature and humidity on TSD estimates.

Recent reports have described the possibility of using mRNA and/or rRNA degradation as a TSD estimator [16], [26]. One study used a semi-quantitative competitive real-time PCR method to evaluate the extent of RNA degradation in bloodstains over time and made the as-yet- unsupported assumption with their assay design that degradation to mRNA occurs in the *ex vivo* dried state in a similar manner to that in the *in vivo* state, namely from the 5′end [26]. Moreover the authors did not attempt to validate their conclusions using dried biological stains exposed to the variety of common environmental insults experienced by real world forensic samples. This study also reported that 4–5 years was required to detect sufficient RNA degradation to distinguish those samples from those deposited earlier, thus limiting the usefulness of this approach. Another study (by different investigators) examined the relative quantities of β-actin mRNA and 18S rRNA as a function of time using real-time PCR [16]. While this latter method has potential applicability in that it is compatible with the current capabilities of most forensic laboratories, it also demonstrated restricted TSD resolution in that samples could only be differentiated, at best, if they were deposited four weeks apart. The β-actin/18S rRNA ratio was calculated using threshold measurements (Ct) obtained by real time PCR but no evidence was adduced as to the extent or type of degradation occurring to the RNA species in support of the authors' assumptions of differential degradation of the two types of RNA molecule.

In an attempt to achieve a better TSD estimate of dried bloodstains than previously reported we examined at high resolution the complete UV-visible absorption spectral profile of Hb in dried bloodstains of different ages. A blue shift (shift to shorter wavelengths) of the Hb Soret band was observed that demonstrated a high correlation with TSD. The extent of this shift permits a distinction to be made between stains that were deposited minutes, hours, days and weeks prior to recovery and analysis.

## Methods

### Preparation of Blood Stains

Peripheral blood samples were collected from volunteers using procedures approved by the University of Central Florida's Institutional Review Board. Informed written consent was obtained from each donor. Blood samples were collected by venipuncture into additive-free vacutainers and 50 µl aliquots were placed onto cotton cloth and dried at room temperature. The bloodstains were then exposed over time to various temperature conditions. Initial studies involved the use of bloodstains placed at room temperature and 37°C protected from light and at ambient humidity for varying lengths of time (15 minutes, 30 minutes, 1 hour, 3 hours, 6 hours, 12–18 hours, 24 hours, 48 hours, 1 week, 1 month, 3 months, 6 months, and 1 year). To assess the effects of humidity, blood samples were collected by venipuncture into additive-free vacutainers and 50 µl aliquots were placed onto non-sterile cotton cloth. The bloodstains were placed in a MicroClimate® Humidity Chamber MCH-3 (Cincinnati Sub-Zero, Cincinnati, OH) at 22°C and 30°C for one week at the following humidity levels: 50%, 75%, 80%, 85%, and 90% humidity. Samples were collected at the following intervals: 15 minutes, 30 minutes, 1 hour, 3 hours, 6 hours, 18 hours, 24 hours, 48 hours and 1 week. In order to assess the effects of temperature, blood samples were collected by venipuncture into additive-free vacutainers and 50 µl aliquots were placed onto non-sterile cotton cloth. These samples were placed at −20°C and 4°C for one week and were collected at the following intervals: 15 minutes, 30 minutes, 1 hour, 3 hours, 6 hours, 18 hours, 24 hours, 48 hours and 1 week. All dried bloodstain samples were stored at −47°C in sealed plastic bags once collected.

### Environmental and Mock Forensic Casework Samples

50 µl aliquots of human blood were dried onto cotton cloth. These samples were exposed to different environmental conditions including heat, light, and humidity for up to 1 week (samples collected at 15 minutes, 30 minutes, 1 hour, 3 hours, 6 hours, 18 hours, 24 hours, 48 hours and 1 week). One set of bloodstains was placed outside on a three-story building rooftop and covered with a glass tank with a mesh bottom to allow exposure to the heat, light and humidity of the outdoor environment. A second set of bloodstains was placed on the floor in the back seat of a car with un-tinted windows. A third set of bloodstains was placed inside the trunk of a car. All samples were stored at −47°C in sealed plastic bags once collected prior to analysis.

### Protein extraction

A 50 µl bloodstain was placed in a 1.5 mL microcentrifuge tube with 750 µl of 0.2 M Tris-HCl, pH 8.0 and incubated overnight at room temperature (protected from light). After incubation, the stains were removed from the supernatant, placed in a spin basket over the supernatant and centrifuged at 14,000 rpm (16,000×g) for 3 minutes. The stains and basket were then discarded. All extracts were stored at −20°C until needed.

### Protein Quantitation

All bloodstain extracts were quantitated using the Quant-It™ Protein Assay Kit (Invitrogen, Carlsbad, CA) according to the manufacturer's recommended conditions. The quantitation was performed using a Synergy™ 2 Microplate Reader (BioTek, Winooski, VT). All samples were run in duplicate and an average of the two measurements obtained.

### UV-Visible Spectroscopy

UV-Visible spectral profiles were obtained using the BioTek Synergy™ 2 Microplate Reader. Spectral data was collected from 200–700 nm in 1 nm increments. Samples were run in a clear, flat-bottomed 96-well reaction plate using 7.5 µg of total protein and brought to a final reaction volume of 75 µl per well using 0.2 M Tris-HCl, pH 8.0. All spectral data was blank corrected using 75 µl of 0.2 M Tris-HCl. For all measurements, bloodstains from two individual donors (one male, one female) were used and an average of the measurements from the two donors reported. All data from the individual donors was run in triplicate and the mean was used for subsequent analysis.

UV-Visible spectral profiles were also obtained from some samples using a U-0080D Photodiode Array Spectrophotometer (Hitachi, Pleasanton, CA). Spectral data was collected from 200–700 nm in 1 nm increments using a 5 µl cell (7.5 µg total protein used for analysis). All spectral data was blank corrected using 5 µl of 0.2 M Tris-HCl. For all measurements, bloodstains from two individual donors (one male, one female) were used and an average of the measurements from the two donors reported. All data from the individual donors was run in triplicate and the mean was used for subsequent analysis.

UV-Visible spectral profiles were also obtained from some samples using the NanoPhotomer™ (Implen, Inc., c/o LABREPCO, Horsman, PA). Spectral data was collected from 350–600 nm in 1 nm increments. Initial evaluations of the instrument were performed using 1 µl aliquots of existing bloodstain extracts from the previous experiments. An optimized protocol for analysis using the NanoPhotometer was developed and included the extraction of 1/4^th^ of a 50 µl bloodstain in 100 µl of 0.2 M Tris-HCl for ∼30 seconds using gentle agitation. For all measurements, bloodstains from two individual donors (one male, one female) were used and an average of the measurements from the two donors is reported.

### Blood Identification

The presence of blood was confirmed with immunochromatography using monoclonal antibodies to human hemoglobin (ABAcard® HemaTrace®, Abacus Diagnostics, West Hills, CA) [27]. Approximately 100 µl of the bloodstain extracts (prepared as described above) were combined with 100 µl of the provided extraction buffer and placed at room temperature for 3 minutes. Four to five drops of the buffer/sample extract were added to the sample well. Results were recorded at 10 minutes. Positive results were indicated by the presence of two pink lines, one in the control region and one in the test region. Negative results were indicated by the presence of only one pink line in the control region.

### DNA Isolation, Quantitation and Autosomal STR Analysis

DNA was isolated from bloodstain extracts prepared as described above using the AllPrep DNA/RNA Mini kit (automated protocol using the QIACube) (Qiagen, Germantown, MD) according to the manufacturer's guidelines. The DNA was eluted into 100 µl of Buffer EB. DNA extracts were quantitated using the Quantifiler™ Human DNA Quantification kit (Applied Biosystems, Foster City, CA) according to manufacturer's protocols. All quantitation assays were performed on a 7500 Real-Time PCR System (Applied Biosystems). One nanogram of DNA was amplified using the PowerPlex® 16 HS System (Promega, Madison, WI) according to the manufacturer's protocol. All amplifications were performed on a GeneAmp® PCR System 9700 thermocycler. A 1.0 µl aliquot of the amplified product was added to 9.5 µl of deionized formamide (Applied Biosystems) and 0.5 µl of ILS-600 internal lane standard (Promega). Samples were injected onto an Applied Biosystems 3130 Genetic Analyzer, using Module F and were analyzed with GeneMapper® Analysis Software v4.0. A peak detection threshold of 150 RFUs was used for allele designation.

## Results

### Ultraviolet-Visible (UV-VIS) Spectroscopy of Hemoglobin in Dried Bloodstains

**Figure 1:**
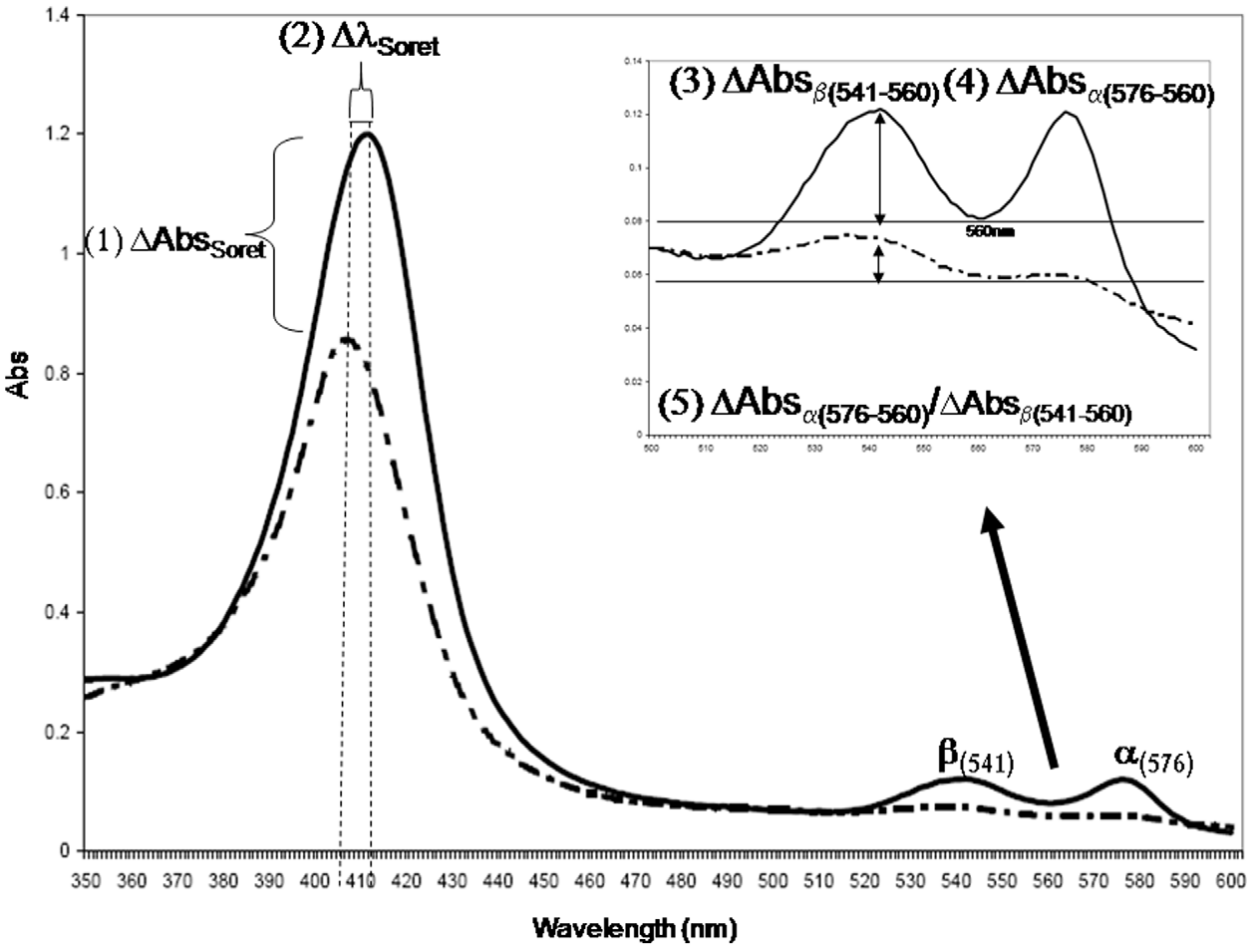
Hemoglobin UV-VIS spectral shift parameters. UV-VIS spectral profiles from bloodstains stored at room temperature for 15 minutes (solid line) and 1 year (dashed line) were compared in order to identify potential differences between “fresh” and “old” stains. The putative time-dependent parameters (1–5) involving changes in the Soret band and α and β peaks are indicated (1–5).

Preliminary studies were carried out to determine whether, at high resolution and across the broad UV-VIS spectrum (200–600 nm), any potentially diagnostic age-related changes to the absorption spectrum of Hb could be discerned in dried bloodstains. In addition to the expected Fe(II) to Fe(III) oxidation of the heme-bound iron, other physico-chemical changes to the Hb molecule such as loss of the water of hydration and partial protein denaturation might also produce subtle effects on the UV-VIS spectrum over time. Initial studies were conducted using a limited number of dried bloodstains on cotton cloth stored at room temperature for a variety of different time periods (15 minutes-1 year). Each sample's UV-VIS absorption spectrum was determined and potential age related changes noted. The spectral parameters that appeared to show some degree of age related changes included: 1) changes in the maximum absorbance of the Soret band (ΔAbs_Soret_); 2) changes in the wavelength of the λmax for the Soret band (Δλmax_Soret_); 3) changes in the relative absorbance of the β band at 541 nm, compared to the λmin at 560 nm (ΔAbs_β(541–560)_); 4) changes in the relative absorbance of the α band at 576 nm, compared to the λmin at 560 nm (ΔAbs _α(576–560)_); 5) the ratio of absorbance changes of the α and β bands (ΔAbs _β(541–560)_/ ΔAbs_α(576–560)_) (Fig. 1). Previous studies on Hb spectral changes with age have concentrated on the α_576_ and β_541_ bands [17]–[19], [21]–[24]. However, our preliminary studies indicated that neither changes to the absorption values of the α_576_ and β_541_ bands (or their ratios) nor the Soret band provided a sufficiently high correlation with age to be a useful TSD diagnostic (data not shown). In contrast the λmax_Soret_ parameter appeared to correlate significantly with age in that a pronounced blue shift (shift to shorter wavelengths) was observed.

**Figure 2:**
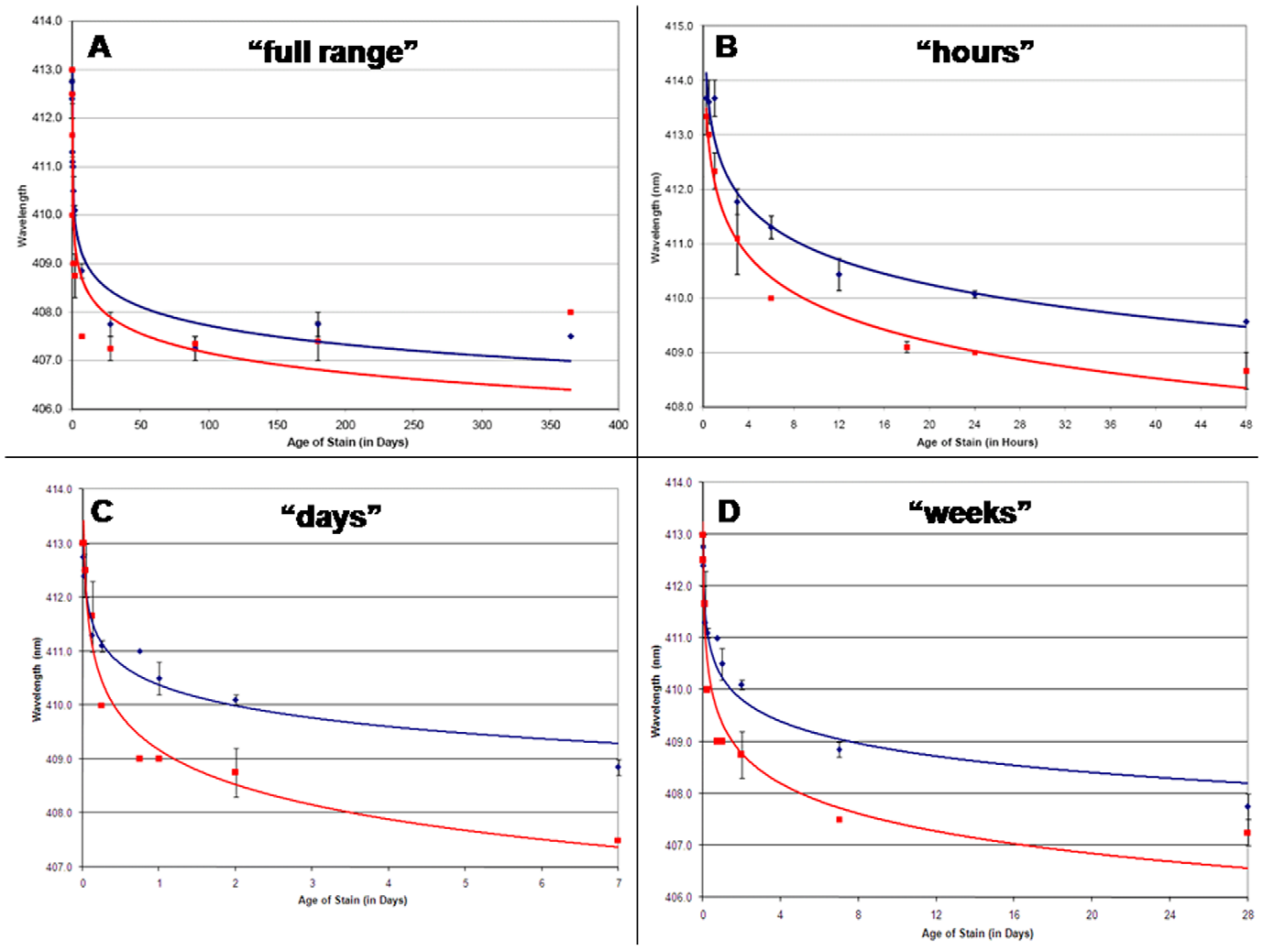
Bloodstain age (TSD) correlates with a blue shift of the Hb Soret band. Spectral profiles (350–600 nm) were obtained from samples stored at 22°C (blue diamonds) and 37°C (red squares) for 15 minutes to 1 year. The λmax_Soret_ is plotted as a function of stain age: A) “full range”, 15 minutes –1 year (in days); B) “hours”, 15 minutes –48 hours (in hours); C) “days”, 15 minutes –7 days (in days); D). “weeks”, 15 minutes –1 month (in days) All data points are an average of bloodstains from two biological replicates (two individual donors). The value used for each of the two biological samples was obtained from the average of three technical replicates for each sample. Using a logarithmic regression function (22°C – blue; 37°C - red), a strong degree of correlation was observed (R^2^>0.95 for most data sets) allowing for differentiation of deposited hours, days, weeks and months prior to collection and analysis. (For interpretation of the references to color in this figure legend, the reader is referred to the web version of the article).

The λmax_Soret_ was graphed as function of stain age for bloodstains (two biological and three technical replicates) stored at both room temperature and at 37°C for 15 minutes to 1 year (Fig. 2A). A negative logarithmic regression function was the best fit to the data. (R^2^ = 0.96 and 0.84 for the 22°C and 37°C samples, respectively, see Table S1). The regression function has the form y = −aLn(x) +410, with y = Soret band (in nm), a = an environmental coefficient the value of which is dependent upon relative humidity and temperature, and x = time (in hours or days). The 37°C samples showed a sharper λmax_Soret_ decrease with early time point samples than the 22°C samples suggesting a possible effect of temperature on the shift. Figure 2B (‘hours’) shows a higher resolution graph that displays bloodstains deposited 15 minutes to 2 days prior to analysis. With this more restricted time frame, the negative logarithmic regression function model also held (R^2^ values of 0.96 and 0.98 for 22°C and 37°C samples, respectively, see Table S1). Other restricted time intervals (15 minutes –1 week (Fig. 2C, ‘days’) and 15 minutes –1 month (Fig. 2D, ‘weeks’)) were also graphed. Collectively the graphs (Figs. 2A–D) depict samples deposited minutes-hours, days, weeks and months before recovery and indicate that such samples might be readily differentiated by λmax_Soret_ measurements. The R^2^ value for each of the four time intervals for both temperatures (except for the 15 min –1 year interval for the 37°C samples) was ≥0.95 (Table S1). It is noted that the lower R^2^ value (0.84) was obtained from the sample set exposed to the highest temperature for the longest period of time 15 min –1 year 37°C and may indicate additional structural changes to the Hb molecule. In a practical sense it is unlikely that bloodstains encountered in the field would be continuously exposed to this high a temperature (∼98°C) over such a prolonged period except perhaps in rare cases.

### Affects of Temperature and Humidity on the Blue Shift of λmax_Soret_

The affects of temperature and humidity on λmax_Soret_ over time were investigated. The initial studies described above used samples that were subjected to ambient room temperature and humidity or to 37°C in an incubator with ambient humidity. In order to more accurately control the temperature and humidity to which the samples were exposed, a digitally controlled and monitored humidity chamber was used. Sets of bloodstains were placed in the humidity chamber at 22°C or 30°C and were exposed to a range of humidity levels from 50–90% (50, 75, 80, 85 and 90%) over a seven day period (168 hours).

#### Humidity

**Figure 3:**
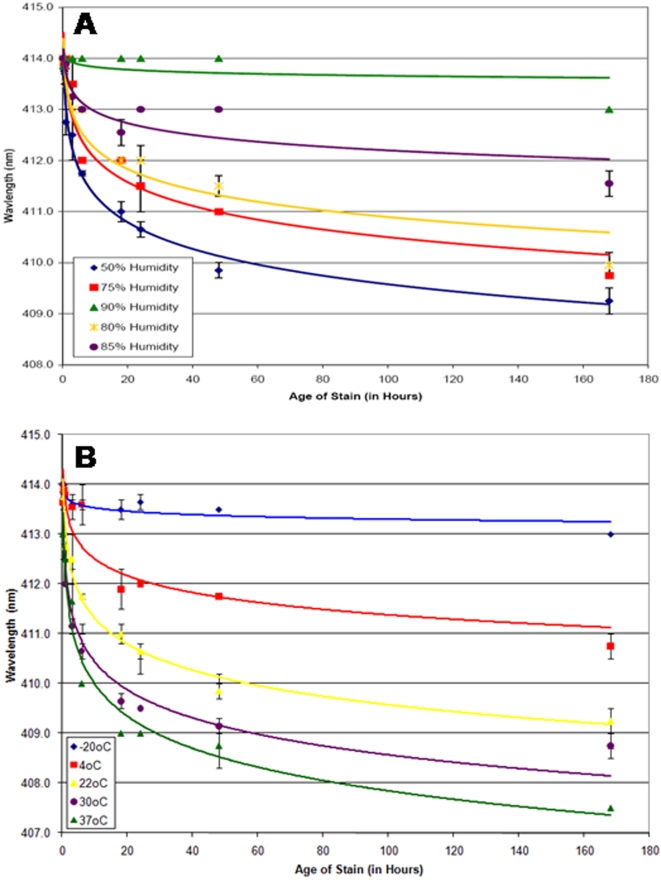
Effects of temperature and humidity on λmax_Soret_ measurements. To assess the effects of humidity, bloodstains were stored at 22°C (A) at various relative humidity (50% - blue diamonds, 75%-red squares, 80%-yellow asterisks, 85%-purple circles, 90%-green diamonds). For bloodstains stored at constant temperature the Soret band blue shift decreases as the humidity level increases. To assess the effects of temperature, bloodstains were stored at a constant relative humidity of 50% (C) at different temperatures (−20°C-blue diamonds, 4°C–red squares, 22°C–yellow asterisks, 30°C-purple circles, 37°C-green triangles). At constant humidity the Soret band blue shift increases with increasing temperature. For each condition, the λmax_Soret_ is plotted as a function of stain age (15 minutes –1 week, in hours). All data points are an average of bloodstains from two individual donors (average of triplicate measurements) and the standard error for each measurement is displayed. (For interpretation of the references to color in this figure legend, the reader is referred to the web version of the article).

The effects of relative humidity levels on the λmax_Soret_ measurements for bloodstains stored at 22°C can be seen in Fig. 3A and in Table 1. As the humidity level increases from 50% to 85% the blue shift decreases from an average rate of 0.028 nm/hour to 0.014 nm/hour over the seven days and, at 90% humidity, is practically non-existent. The affects of humidity on samples stored at 30°C was also tested (data not shown). Again, as the humidity level increases, the blue shift decreases (data not shown). The effects of humidity are different depending on what temperature the sample is exposed to. For example, for bloodstains stored at 22°C, there is a progressive reduction in the blue shift as the humidity level increases (Fig. 3A). It was expected that the same trend would be observed for the bloodstains stored at 30°C. However, the extent of the blue shift with 50% and 75% humidity is quite similar (data not shown). Therefore it is possible that at higher temperatures the affect of humidity is lessened and a higher humidity level is needed to cause a reduction in the blue shift.

**Table 1:** Effects of Temperature and Humidity on the Soret Band Blue Shift.

	Humidity (%)	Average Blue Shift Rate (nm/hour)	R^2^	Regression function equation
	90	0.005	0.2473	y = −0.075Ln(x) +414
	85	0.014	0.8422	y = −0.3308Ln(x) +413.72
22°C	80	0.024	0.9351	y = −0.5826Ln(x) +413.58
	75	0.025	0.928	y = −0.6685Ln(x) +413.58
	50	0.028	0.9861	y = −0.7548Ln(x) +413.05

	Temperature (°C)	Average Blue Shift Rate (nm/hour)	R^2^	Regression function equation
	−20	0.005	0.4533	y = −0.111Ln(x) +413.68
	4	0.017	0.8495	y = −0.4899Ln(x) +413.63
50%	22	0.028	0.9861	y = −0.7548Ln(x) +413.05
	30	0.031	0.9602	y = −0.8137Ln(x) +412.31
	37	0.033	0.9664	y = −0.9322Ln(x) +412.13

#### Temperature

The above experiments examined the effects of different humidity levels at a constant temperature. Conversely, the effects of temperature at a constant humidity were also studied. Data for bloodstains stored at −20°C, 4°C, 22°C, 30°C and 37°C (each at 50% humidity) was obtained (Table 1). The λmax_Soret_ was plotted for each temperature as a function of stain age (Fig. 3B). The blue shift was larger and occurred at a faster rate with increased temperature. For example, at 37°C the average blue shift rate was 0.033 nm/hour whereas the rate decreased to 0.017 nm/hour at 4°C. Of additional practical interest (see below) was the −20°C sample set where no significant change in the λmax_Soret_ was observed over time.

### Assay Validation

#### Recovery and Storage of Bloodstain Samples for λmax_Soret_ Analysis

The temperature data suggested that short term laboratory storage at −20°C of bloodstains removed from a crime scene may halt post-collection λmax_Soret_ changes. In order to further test the ability to store bloodstains at −20°C without affecting λmax_Soret_ measurements, bloodstain samples were subjected to four different conditions prior to analysis: 1) samples were analyzed immediately upon collection, 2) samples were collected and stored at −20°C for two weeks before analysis, 3) samples collected and stored at −20°C for four weeks before analysis and 4) samples were recovered and stored at −47°C until the last one (1 week) was collected and then all samples were analyzed (Fig. S1A). Similar λmax_Soret_ plots were obtained for each of the conditions described above indicating that it may be possible for bloodstains to be collected at a crime scene and stored frozen (−20°C) for several weeks without adversely impacting the accuracy of the analysis. However long term storage of samples at −47°C for 8 months prior to analysis resulted in a decrease in the R^2^ value (from 0.96 to 0.86) and an increase in the blue shift compared to samples analyzed within two weeks of collection (Fig. S1B).

#### Sensitivity

The initial development work with the assay used 0.1 mg/ml of total protein (7.5 µg in 75 µl of buffer) with a minimum of 2.2 µg of total protein being needed to obtain the diagnostic blood-specific Hb α and β bands (in addition to the Soret band) (data not shown). In order to further test the practical sensitivity of the method bloodstains ranging in size from 0.2–10 µl were prepared (Fig. S2A). Analysis of bloodstains <1 µl in size resulted in poor recovery of α_576_ and β_541_ bands, and the Soret band was too broad for an accurate determination of the λmax_Soret_. Therefore, various volumes of the 1 µl bloodstain (from a 25 µl extract in Tris buffer) were tested ranging from 0.25–3 µl (equivalent to 10–120 nl of liquid blood). The Soret band was visible down to 0.5 µl of the extract (20 nl liquid blood equivalent) (Fig. S2B) but required at least 2–3 µl (80–120 nl liquid blood equivalent) to obtain the diagnostic Hb α_576_ and β_541_ bands (Fig. S2C).

While it was necessary to demonstrate the ability to obtain accurate and reliable λmax_Soret_ measurements from small bloodstains, it was equally as important to demonstrate that these measurements would not be affected by significant larger bloodstains. Large bloodstains or pools of blood can frequently be encountered at crime scenes. The outer regions of the bloodstain will dry quicker than the center of the bloodstain resulting in capillary action separation of soluble constituents and, conceivably, the different regions of the stain might be exposed to slightly different degradative processes. Therefore, the λmax_Soret_ was determined in 60 µl bloodstains and compared to that of a 600 µl bloodstain from the same donor and prepared at the same time. The large 600 µl bloodstain had a lighter colored outer portion with a denser center portion (Fig. S3A). Samples from the outer and central portions of the 600 µl bloodstain were collected and the Δλmax_Soret_ determined for each. The size of the bloodstain or the location of the sampling did not affect the blue shift (Fig. S3B) indicating that crime scene analysts may be able to collect samplings from different regions of deposited bloodstains.

#### Environmental Exposure

The previously described experiments examined the affects of storage at constant temperatures and humidity levels. However, some criminal offenses will not occur indoors in a controlled environment with relatively stable temperatures and humidity levels. Often crime scenes are located outside with samples exposed to fluctuating temperatures and humidity levels, sunlight, moisture, bacterial growth, and possible smog or other air pollutants. Therefore, it was prudent to examine bloodstains that were stored outside exposed to the natural elements. Bloodstains were placed outside and covered (OSC), exposed to the natural fluctuations in temperature and humidity but protected from rain and collected at various times through a one week period (15 minutes, 30 minutes, 1 hour, 3 hours, 6 hours, 18 hours, 24 hours, 48 hours and 1 week). During this week period the samples were exposed to a reported average temperature of 27.1°C (low 22.7°C, high 35.7°C) and an average humidity of 81.1% (low 46%, high 97.9%) (Table S2). The λmax_Soret_ for the OSC samples was plotted as a function of stain age (Fig. S4). The extent of the blue shift was greater for the OSC samples compared to other data sets exposed to high temperature (37°C shown in Fig. S4 for comparison). It might have been expected that the blue shift would have been less than that of the 37°C due to the reported average temperature (27.1°C) and reported average high and low temperatures (22.7°C–35.7°C) that the OSC samples had been exposed to. However, while the average high temperature for the OSC data set was 35.7°C, there were three days where the recorded high temperature was greater than 37°C (38.1°C–39.4°C) and since the bloodstains were exposed to direct sunlight at various times during the day, it is possible that they experienced temperatures much higher than the reported temperatures (which are recorded in the shade). Without a direct and continuous measurement of the actual temperature, the reported average temperature and humidity levels can only be used as a crude approximation of the actual temperatures. It is also possible that sunlight or air pollutants could also contribute to an increase in the blue shift of samples exposed to outside environmental conditions. However, as can be seen in Fig. S4, a distinction can still be made between bloodstain samples that were minutes, hours and days old using the λmax_Soret_ measurement.

To further examine the affects of heat and humidity using samples more akin to those encountered in casework, bloodstains were stored inside the trunk of a car and on the floor in the back seat of a car for various lengths of time ranging from 25 minutes to 1 week, including 25 minutes, 1 hour, 3 hours, 18 hours, 24 hours, 48 hours, 5 days (112 hours), and 1 week (Fig. S4). A digital thermometer with humidity gauge was placed inside the car trunk and in the back seat to allow for monitoring of temperature and humidity. The temperature and humidity levels were recorded at each sample collection interval. During the week period, the bloodstains in the car trunk were exposed to an average temperature of 42.7°C (high 44.6°C, low 43.4°C) and an average relative humidity of 50% (high 60%, low 40%). During the week period, the bloodstains in the back seat were exposed to an average temperature of 41.9°C (high 44.9°C, low 39.4°C) and an average relative humidity of 51% (high 57%, low 40%). Due to the extreme temperature, a larger blue shift compared to previous data sets (up to 37°C samples) was expected. As can be seen from Fig. S4, the extent of the blue shift for the car samples was between that of the OSC and 37°C samples. Again, a clear distinction between samples differing in age by minutes, hours and days can be made. However, this data also confirms that a good estimate of the exposure conditions would be critical to the accuracy of TSD estimation.

### Instrumentation

#### Bench-top Spectrophotometer

The previous data were obtained using a particular benchtop spectrophotometer (BioTek Synergy 2 microplate reader). In order to assess the degree to which the blue Soret band shift was instrument specific, an evaluation of a duplicate set of bloodstains was performed on another manufacturer's instrument (Hitachi U-0080D Spectrophotometer). The λmax_Soret_ for bloodstains stored at 22°C, 50% humidity (15 minutes–1 week) was obtained using both instruments and plotted as a function of stain age (data not shown). The blue shift was similar with both instruments (data not shown). There were, however, differences in the obtained wavelength values between the two spectrophotometers, with the values for the Hitachi spectrophotometer typically 0.7–1.7 nm higher (average = 1.1 nm) than observed for the Synergy™ 2 microplate reader. The total overall wavelength shift between the 15 minute and 1 year samples was generally the same between the two instruments for both data sets. The R^2^ for the 22°C data sets on both instruments was greater than 0.91 (data not shown). The same trend was observed in other sample set data from both instruments, with the λmax_Soret_ from the Hitachi spectrophotometer slightly higher than the BioTek microplate reader (data not shown). Thus the observed blue shift in aged bloodstains appears to be a genuine phenomenon but that there is a need to pre-calibrate any spectrophotometer to be used for TSD measurements. The microplate reader offers the advantage of being able to run replicates of the same sample at the same time, as well as the ability to run more samples at one time (96-well plate vs. a single cuvette with the spectrophotometer) which would be useful for routine forensic casework. It also uses a disposable 96-well plate whereas a standard bench-top spectrophotometer would require the re-use of a cuvette which would need to be cleaned between samples in order to avoid potential contamination.

#### Portable ‘Point-Of-Use’ Spectrophotometer

We have demonstrated that λmax_Soret_ measurements and characteristic blood spectral peaks can be obtained from bloodstains as small as 1 µl. The ability to perform spectroscopic measurements at the scene could make the technique even more appealing to forensic investigators since one could in principle positively identify suspected bloodstains *in situ* as well as estimate how old the stain was. Accordingly we evaluated a portable spectrophotometer (Implen NanoPhotometer™) that has no moving parts making it more durable during transport to a crime scene, weighs less than 10 lbs, permits full wavelength scans (190–1100 nm) and, importantly, uses only 0.7 µl of buffer-extracted sample.

Bloodstains stored at 22°C (50% and 90% humidity), 30°C (50% and 90% humidity) and the samples placed on the floor of the back seat of a car were analyzed using the NanoPhotometer™. Similar λmax_Soret_ values and linear regression functions were obtained for the samples using both NanoPhotometer™ and the Synergy™ 2 microplate instruments (data not shown). Significantly, only slight variation in the λmax_Soret_ values for the samples placed on the floor in the back seat of a car (except for the 48 hour sample with a 1 nm difference) were observed. The R^2^ value for both instruments was >0.9. The larger differences between instruments for some of the data points for the other exposure conditions (data not shown) could be due to the greater length of time that the 22°C and 30°C bloodstains were stored prior to re-analysis on the NanoPhotometer™. Although the initial data indicates the potential utility of a portable instrument, additional validation studies involving an evaluation of actual casework samples with the portable instrument of choice would be required.

### DNA Isolation and Typing of Bloodstain Extracts used for TSD measurements

**Figure 4:**
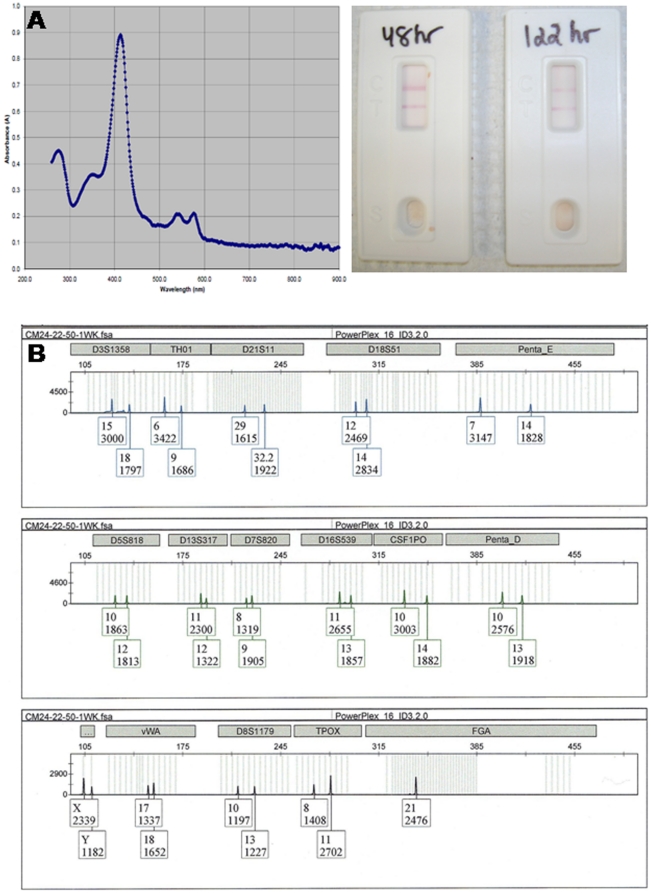
TSD sample extracts can be used to confirm the presence of human blood and obtain the DNA profile of the bloodstain donor. The use of a portable spectrophotometer (NanoPhotometer™) permits the rapid identification of the presence of blood via its characteristic Hb absorption spectrum (A, left panel). Further confirmation of the presence of blood and its human origin was provided by a positive result with an immunochromatographic test (ABAcard® HemaTrace®) (A, right panel) using an aliquot of the same TSD extract. DNA was isolated from the remaining extract and a full autosomal DNA profile (PowerPlex® 16 HS, Promega) was obtained using standard amplification conditions (B).

We performed serological and genetic analysis on the same bloodstain extracts used for TSD analysis. The recovery of the characteristic blood Hb spectral peaks (Soret band, α_576_ and β_541_ bands) permits identification of the presence of blood (Fig. 4A, left panel) but provides no indication of the species of origin. An aliquot of the bloodstain extract used for TSD measurements was tested using an immunochromatographical method employing monoclonal antibodies to human hemoglobin. Positive results for human blood were obtained for a set of bloodstains from eleven individuals that had been exposed to 22°C and 50% humidity for up to 1 week (Fig. 4A, right panel). The remaining portion of the original bloodstain extract was used to isolate DNA for DNA profiling using autosomal STRs in order to identify the donor of the bloodstain. Varying amounts of DNA were recovered from the bloodstains tested (Table S3), but all eleven samples resulted in the recovery of complete autosomal STR profiles (Fig. 4B).

## Discussion

One of the principal raisons d'être of forensic science is to try and establish a fact that may be at issue. Standard DNA profiling is able to identify the donor of a bloodstain deposited at the crime scene but is unable to provide an indication as to when the blood was deposited. We believe that the preliminary work reported here could form the foundation of a robust TSD estimator that would aid crime scene investigations. We report a blue shift in the visible absorption spectrum of the Hb Soret band (λ max = 410 nm) that strongly correlates with the age of the bloodstain. The magnitude of the shift (0–7 nm) is dependent upon the ambient temperature and relative humidity. The data fit an empirical negative logarithm function well, as indicated by high R^2^ values and this mathematical model might therefore be ‘fit for purpose’ in its present form (i.e. sufficiently accurate and pragmatic for field use). However alternative mathematical models that better describe the kinetics of the blue shift may exist and will be the subject of future enquiry. The molecular basis for the observed Soret band blue shift is likely based upon the well characterized oxidation chemistry and spectroscopy of Hb [28]–[32]. Thus oxyHb in the ferrous state is first converted to the high spin ferric protein, methemoglobin (metHb). Over time metHb is converted, both reversibly and irreversibly, to hemi-chromes which are low spin ferrihemoglobin derivatives comprising protein conformational changes in which endogenous metHb protein atoms are coordinated to the sixth ligand of the heme iron. The order of events is almost certainly oxidation, dehydration and hemichrome formation followed by the eventual loss of coordinated heme and release of free iron. Interestingly, however, in the dried state oxidative changes appear to be reduced and there is some evidence for the formation of additional denatured non-hemichrome Hb derivatives [33].

The potential utility of the Soret band blue shift in forensic casework is promising. This is due to a number of factors including good time resolution (bloodstains deposited hours, days, and weeks can be distinguished), sensitivity (1 µl bloodstains) and the potential to perform this analysis at the crime scene through the use of a portable ‘point-of-use’ spectrophotometer. As a corollary, the latter could also be used to identify the presence of blood *per se* due to the characteristic absorption spectrum of Hb with its Soret and α_576_ and β_541_ bands. We have also demonstrated the ability to perform serological testing and DNA profiling on a single TSD extract without the necessity to consume additional sample. Of practical consequence is the observation that the blue shift of the Soret band is more or less abolished at ≤−20°C at least over a several week period. Thus crime scene investigators can recover the samples and immediately store them at −20°C until it is convenient for the TSD analysis to proceed. A laboratory seeking to implement the TSD method for casework would have to extensively validate the method in their laboratory. Specifically they would have to calibrate a particular spectrophotometer using bloodstains of different ages from a number of different individuals and subject them to geographically relevant different temperatures and humidities as well as to any other commonly found environmental insults (e.g. terrestrial radiation from the sun). It is also recommended that blind testing be performed in which the analyst is provided a set of bloodstains deposited at different (but known) times and estimated temperature and humidity conditions. The efficacy of the predictive power of the assay is then determined by comparison of the predicted and expected values. A potential impediment to the implementation of the λmax_Soret_ method is its temperature and humidity dependence. This requires estimation by the analyst of the environmental conditions experienced by the bloodstain since its deposition. Of course, a precise knowledge of this would require that the age of the stain is known, the variable that is trying to be determined by the analysis. Although bloodstains deposited outdoors will be exposed to different temperatures and humidities dependent upon the geographical location and the season, many locations will have a finite and restricted range of possible values. According to National statistics, for example, most of the US experiences humidity levels between 56 and 85% for most of the year with Western states experiencing lower humidity levels (35–50%) for most of the year [34]. Therefore different regions of the country will be exposed to different humidity and temperature throughout the seasons. In some states, such as Arizona or New Mexico, humidity levels >60% might not be relevant whereas levels <65% might not be particularly relevant for states such as Florida. It would be incumbent upon the analyst to estimate the expected ranges of the temperatures and humidities the bloodstain was likely exposed to and apply the appropriate confidence intervals around the expected λmax_Soret_ values. Many crimes take place indoors where the temperature and humidity is precisely controlled and easily estimated (or measured) thus making estimation of TSD more facile.

## Supporting Information

Table S1 Soret Band Blue Shift with Age of Bloodstain Follows a Negative Logarithmic Function.
(0.04 MB DOC)

Table S2 Summary of Temperature and Precipitation Conditions for Bloodstains Stored Outside (1 week).
(0.05 MB DOC)

Table S3 DNA Recovery and STR Typing from TSD Bloodstain Extracts.
(0.04 MB DOC)

Figure S1 Effects of sample storage prior to λmax_Soret_ measurements. A) A set of bloodstains was exposed to 22°C, 50% humidity for 15 minutes to 1 week. A portion of the bloodstain was tested immediately upon collection of the sample (red squares), while the remaining portions were stored at −20°C in sealed plastic bags and tested after 2 weeks (yellow circles) and 4 weeks (green triangles) of storage. The resulting λmax_Soret_ was plotted for each storage condition and compared to the original data set (22°C, 50% humidity) in which samples were stored at −20°C until the one week sample was collected (blue diamonds). R^2^ values were similar regardless of the length of short-term storage. B) A set of bloodstains stored at 22°C, 50% humidity for 15 minutes to 1 week was retested after storage at −47°C for 8 months. Storage of bloodstains for the 8 month period resulted in a weaker correlation (R^2^ = 0.86) compared to the original test data developed after 0–4 weeks storage prior to analysis.
(0.18 MB TIF)

Figure S2 Determination of the sensitivity of the blue shift assay - bloodstain size. In order to determine if accurate TSD measurements could be made with small bloodstains, 0.2 µl to 10 µl bloodstains (A) were placed on cotton cloth and dried overnight. The bloodstains were extracted in 25 µl and 50 µl of 0.2 M Tris-HCl buffer. The blue shift (B) could be observed using as little as 20 nanoliters of blood (from a 1 µl bloodstain). However, to obtain a characteristic blood spectral profile 80–120 nl of blood (from a 1 µl stain) is required (C).
(0.42 MB TIF)

Figure S3 Comparison of λmax_Soret_ measurements from small (∼60 µl) and large (∼600 µl) bloodstains. Samples previously tested consisted of 50 µl bloodstains. However, often larger “pools” of blood may be encountered at crime scenes. The outer potions of larger bloodstains will dry at different rates than the larger volume in the center of the bloodstain. In order to determine if the unequal drying times would affect the accuracy of TSD measurements, 600 µl (A, left -panel) and 60 µl (A, right panel) bloodstains were placed at 22°C, 50% humidity for 15 minutes to 1 week. Portions of the 600 µl taken from the center and outer edge (A, yellow squares) were analyzed. B). Similar λmax_Soret_ values were observed for all samples regardless of the original stain size or sampling location.
(0.44 MB TIF)

Figure S4 Blue shift of the Hb Soret band in bloodstains exposed to the environment. Bloodstains on cotton cloth were placed outside, covered with exposure to heat, light and humidity (red squares), on the floor in the back seat of a car with non-tinted windows (green circles), and in the trunk of a car (blue diamonds). As a control a bloodstain was stored in the laboratory at 37°C at 50% relative humidity (yellow triangles). Samples were collected at various intervals within 15 minutes to 1 week of exposure. The λmax_Soret_ is plotted as a function of stain age (15 minutes –1 week, in hours). Also shown are the associated logarithmic regression functions and correlation coeficients (R^2^ value). All data points are an average of bloodstains from two individual donors (average of triplicate measurements) and the standard error for each measurement is displayed. (For interpretation of the references to color in this figure legend, the reader is referred to the web version of the article).
(0.10 MB TIF)
