# Supplementary material for: A Blue Spectral Shift of the Hemoglobin Soret Band Correlates with the Age (Time Since Deposition) of Dried Bloodstains
Source: PLoS One. 2010 Sep 20;5(9):e12830. doi: 10.1371/journal.pone.0012830 (PMC2942901; doi:10.1371/journal.pone.0012830)
Supplement: Table S1 — Soret Band Blue Shift with Age of Bloodstain Follows a Negative Logarithmic Function. (0.04 MB DOC) [file pone.0012830.s001.doc]

|  | **Temperature (^o^C)** | **R^2^** | **Regression function equation** |
| --- | --- | --- | --- |
| **Hours** | 22 | 0.955 | y = -0.886Ln(x) + 412.9 |
|  | 37 | 0.9843 | y = -0.9767Ln(x) + 412.13 |
| **Days** | 22 | 0.9481 | y = -0.5571Ln(x) + 410.37 |
|  | 37 | 0.9667 | y = -0.9251Ln(x) + 409.17 |
| **Weeks** | 22 | 0.9566 | y = -0.6121Ln(x) + 410.24 |
|  | 37 | 0.9556 | y = -0.8413Ln(x) + 409.36 |
| **Full Range** | 22 | 0.9626 | y = -0.5647Ln(x) + 410.32 |
|  | 37 | 0.843 | y = -0.5837Ln(x) + 409.84 |
