# Supplementary material for: A Blue Spectral Shift of the Hemoglobin Soret Band Correlates with the Age (Time Since Deposition) of Dried Bloodstains
Source: PLoS One. 2010 Sep 20;5(9):e12830. doi: 10.1371/journal.pone.0012830 (PMC2942901; doi:10.1371/journal.pone.0012830)
Supplement: Table S2 — Summary of Temperature and Precipitation Conditions for Bloodstains Stored Outside (1 week). (0.05 MB DOC) [file pone.0012830.s002.doc]

|  | **Temperature (^o^F)** | | | **Temperature (^o^C)** | | | **Relative Humidity (%)** | | |
| --- | --- | --- | --- | --- | --- | --- | --- | --- | --- |
|  | **High** | **Low** | **Avg** | **High** | **Low** | **Avg** | **High** | **Low** | **Avg** |
| **Day 0** | 92.8 | 73.2 | 77.3 | 33.8 | 22.9 | 25.2 | 98 | 56 | 91 |
| **Day 1** | 87.4 | 73.0 | 77.0 | 30.8 | 22.8 | 25.0 | 98 | 65 | 92 |
| **Day 2** | 93.6 | 73.4 | 77.9 | 34.2 | 23.0 | 25.5 | 98 | 50 | 89 |
| **Day 3** | 94.3 | 73.6 | 79.7 | 34.6 | 23.1 | 26.5 | 98 | 52 | 87 |
| **Day 4** | 100.6 | 72.3 | 83.7 | 38.1 | 22.4 | 28.7 | 98 | 35 | 74 |
| **Day 5** | 102.9 | 73.6 | 85.8 | 39.4 | 23.1 | 29.9 | 98 | 30 | 71 |
| **Day 6** | 102.6 | 73.9 | 85.0 | 39.2 | 23.3 | 29.4 | 98 | 28 | 70 |
| **Day 7** | 95.5 | 70.0 | 80.3 | 35.3 | 21.1 | 26.8 | 97 | 51 | 80 |
| **Average** | **96.2** | **72.9** | **80.8** | **35.7** | **22.7** | **27.1** | **97.9** | **45.9** | **81.8** |

*data obtained from <http://www.wunderground.com/weatherstation/> (UCF/Alafaya/Oviedo area - Florida)
