# Supplementary material for: A Blue Spectral Shift of the Hemoglobin Soret Band Correlates with the Age (Time Since Deposition) of Dried Bloodstains
Source: PLoS One. 2010 Sep 20;5(9):e12830. doi: 10.1371/journal.pone.0012830 (PMC2942901; doi:10.1371/journal.pone.0012830)
Supplement: Table S3 — DNA Recovery and STR Typing from TSD Bloodstain Extracts. (0.04 MB DOC) [file pone.0012830.s003.doc]

| **Donor** | **Exposure Condition** | **Length of Exposure** | **Quantity (ng/µl)**  **(Quantifiler Human)** | **Amount Amplified** | **PowerPlex 16 HS** |
| --- | --- | --- | --- | --- | --- |
| **Female** | Car back seat | 1 week | 0.23 | 1.2 ng | Full profile |
| **Female** | 22^o^C, 50% humidity | 1 week | 0.20 | 2.0 ng | Full profile |
| **Male** | 22^o^C, 50% humidity | 1 week | 0.06 | 624 pg | Full profile |
| **Female** | 22^o^C, 80% humidity | 1 week | 0.04 | 446 pg | Full profile |
| **Male** | 22^o^C, 80% humidity | 1 week | 0.02 | 228 pg | Full profile |
| **Female** | 22^o^C, 90% humidity | 1 week | 0.02 | 120 pg | Full profile |
| **Male** | 22^o^C, 90% humidity | 1 week | 0.05 | 485 pg | Full profile |
| **Female** | 30^o^C, 50% humidity | 1 week | 0.02 | 152 pg | Full profile |
| **Male** | 30^o^C, 50% humidity | 1 week | 0.02 | 186 pg | Full profile |
| **Female** | 30^o^C, 90% humidity | 1 week | 0.58 | 2.0 ng | Full profile |
| **Male** | 30^o^C, 90% humidity | 1 week | 0.05 | 456 pg | Full profile |
