# Supplementary figures and images for: A Blue Spectral Shift of the Hemoglobin Soret Band Correlates with the Age (Time Since Deposition) of Dried Bloodstains
Source: PLoS One. 2010 Sep 20;5(9):e12830. doi: 10.1371/journal.pone.0012830 (PMC2942901; doi:10.1371/journal.pone.0012830)

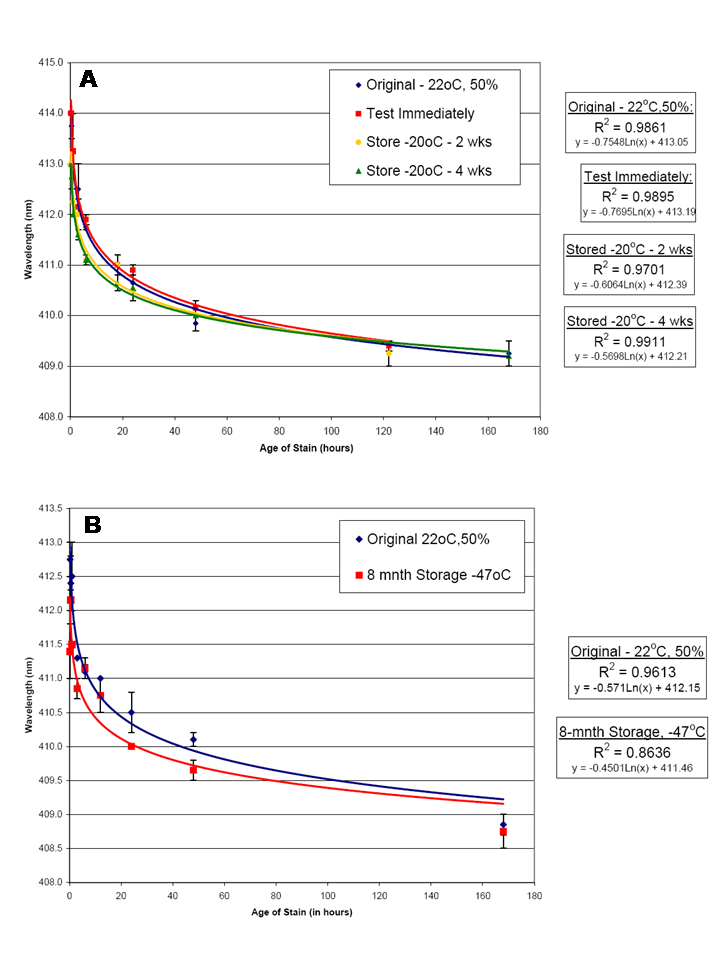

Supplement: Figure S1 — Effects of sample storage prior to λmaxSoret measurements. A) A set of bloodstains was exposed to 22°C, 50% humidity for 15 minutes to 1 week. A portion of the bloodstain was tested immediately upon collection of the sample (red squares), while the remaining portions were stored at −20°C in sealed plastic bags and tested after 2 weeks (yellow circles) and 4 weeks (green triangles) of storage. The resulting λmaxSoret was plotted for each storage condition and compared to the original data set (22°C, 50% humidity) in which samples were stored at −20°C until the one week sample was collected (blue diamonds). R2 values were similar regardless of the length of short-term storage. B) A set of bloodstains stored at 22°C, 50% humidity for 15 minutes to 1 week was retested after storage at −47°C for 8 months. Storage of bloodstains for the 8 month period resulted in a weaker correlation (R2 = 0.86) compared to the original test data developed after 0–4 weeks storage prior to analysis. (0.18 MB TIF) [file pone.0012830.s004.tif]

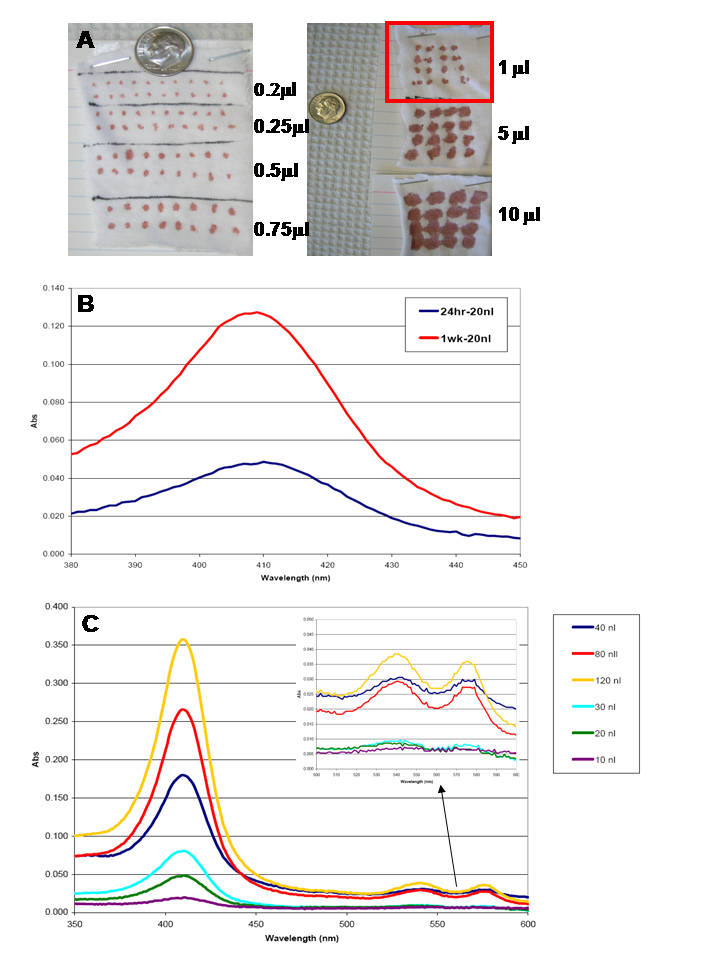

Supplement: Figure S2 — Determination of the sensitivity of the blue shift assay - bloodstain size. In order to determine if accurate TSD measurements could be made with small bloodstains, 0.2 µl to 10 µl bloodstains (A) were placed on cotton cloth and dried overnight. The bloodstains were extracted in 25 µl and 50 µl of 0.2 M Tris-HCl buffer. The blue shift (B) could be observed using as little as 20 nanoliters of blood (from a 1 µl bloodstain). However, to obtain a characteristic blood spectral profile 80–120 nl of blood (from a 1 µl stain) is required (C). (0.42 MB TIF) [file pone.0012830.s005.tif]

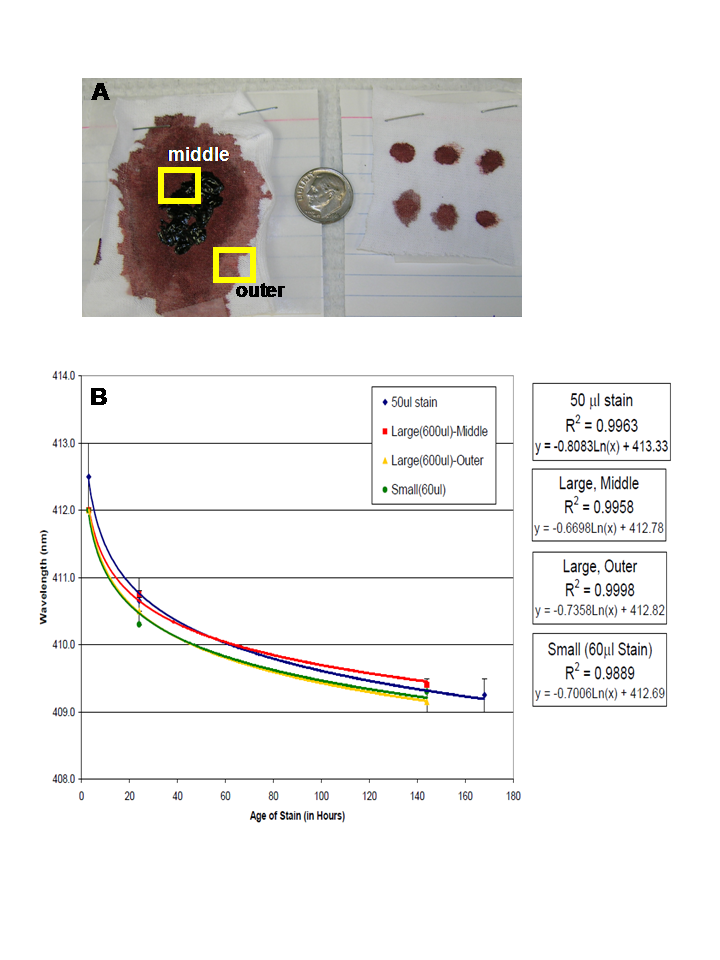

Supplement: Figure S3 — Comparison of λmaxSoret measurements from small (∼60 µl) and large (∼600 µl) bloodstains. Samples previously tested consisted of 50 µl bloodstains. However, often larger “pools” of blood may be encountered at crime scenes. The outer potions of larger bloodstains will dry at different rates than the larger volume in the center of the bloodstain. In order to determine if the unequal drying times would affect the accuracy of TSD measurements, 600 µl (A, left -panel) and 60 µl (A, right panel) bloodstains were placed at 22°C, 50% humidity for 15 minutes to 1 week. Portions of the 600 µl taken from the center and outer edge (A, yellow squares) were analyzed. B). Similar λmaxSoret values were observed for all samples regardless of the original stain size or sampling location. (0.44 MB TIF) [file pone.0012830.s006.tif]

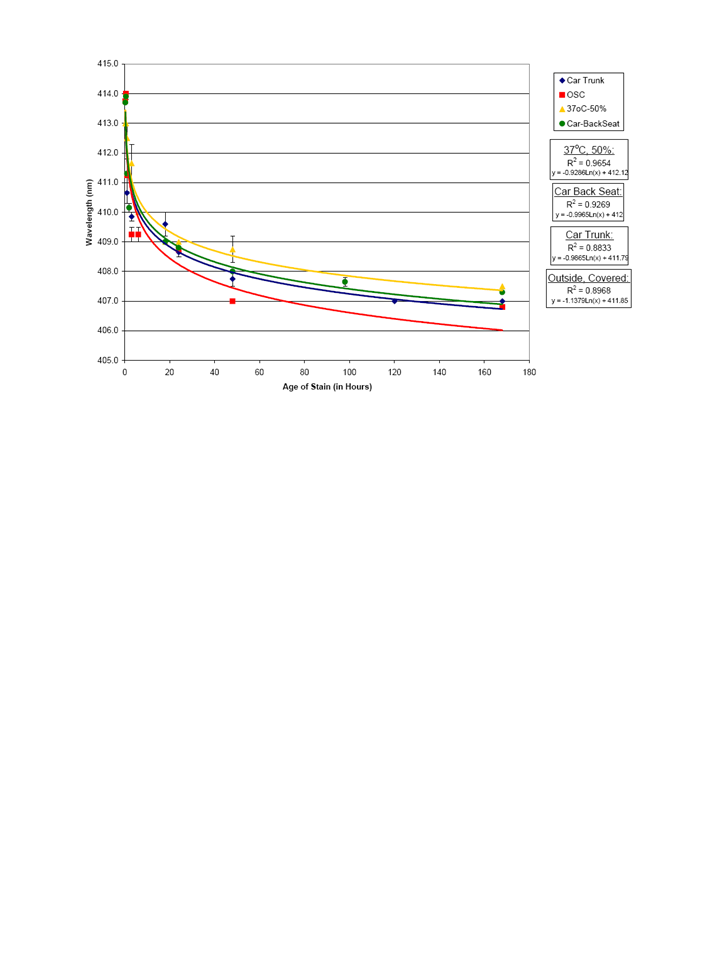

Supplement: Figure S4 — Blue shift of the Hb Soret band in bloodstains exposed to the environment. Bloodstains on cotton cloth were placed outside, covered with exposure to heat, light and humidity (red squares), on the floor in the back seat of a car with non-tinted windows (green circles), and in the trunk of a car (blue diamonds). As a control a bloodstain was stored in the laboratory at 37°C at 50% relative humidity (yellow triangles). Samples were collected at various intervals within 15 minutes to 1 week of exposure. The λmaxSoret is plotted as a function of stain age (15 minutes –1 week, in hours). Also shown are the associated logarithmic regression functions and correlation coeficients (R2 value). All data points are an average of bloodstains from two individual donors (average of triplicate measurements) and the standard error for each measurement is displayed. (For interpretation of the references to color in this figure legend, the reader is referred to the web version of the article). (0.10 MB TIF) [file pone.0012830.s007.tif]
